# Sigma-1 Receptor Antagonist BD1047 Reduces Mechanical Allodynia in a Rat Model of Bone Cancer Pain through the Inhibition of Spinal NR1 Phosphorylation and Microglia Activation

**DOI:** 10.1155/2015/265056

**Published:** 2015-11-30

**Authors:** Shanshan Zhu, Chenchen Wang, Yuan Han, Chao Song, Xueming Hu, Yannan Liu

**Affiliations:** ^1^Department of Anesthesiology, Xuzhou Cancer Hospital, Affiliated Xuzhou Hospital, Jiangsu University, Xuzhou 221005, China; ^2^Jiangsu Province Key Laboratory of Anesthesiology, Xuzhou Medical College, Xuzhou 221002, China; ^3^Jiangsu Province Key Laboratory of Anesthesia and Analgesia Application Technology, Xuzhou 221002, China; ^4^Department of Anesthesiology, Xuzhou Children's Hospital, Xuzhou 221006, China; ^5^Department of Anesthesiology, Affiliated Hospital, Xuzhou Medical College, Xuzhou 221006, China; ^6^Department of Oncology, Affiliated Hospital, Xuzhou Medical College, Xuzhou 221006, China; ^7^Department of Pain, Affiliated Hospital, Xuzhou Medical College, Xuzhou 221006, China; ^8^Department of Anesthesiology, Xuzhou Maternity and Child Health Hospital, Xuzhou 221009, China

## Abstract

Previous studies have demonstrated that sigma-1 receptor plays important roles in the induction phase of rodent neuropathic pain; however, whether it is involved in bone cancer pain (BCP) and the underlying mechanisms remain elusive. The aim of this study was to examine the potential role of the spinal sigma-1 receptor in the development of bone cancer pain. Walker 256 mammary gland carcinoma cells were implanted into the intramedullary space of the right tibia of Sprague-Dawley rats to induce ongoing bone cancer-related pain behaviors; our findings indicated that, on days 7, 10, 14, and 21 after operation, the expression of sigma-1 receptor in the spinal cord was higher in BCP rats compared to the sham rats. Furthermore, intrathecal injection of 120 nmol of sigma-1 receptor antagonist BD1047 on days 5, 6, and 7 after operation attenuated mechanical allodynia as well as the associated induction of c-Fos and activation of microglial cells, NR1, and the subsequent Ca^2+^-dependent signals of BCP rats. These results suggest that sigma-1 receptor is involved in the development of bone cancer pain and that targeting sigma-1 receptor may be a new strategy for the treatment of bone cancer pain.

## 1. Backgrounds

Bone cancer pain (BCP), which is the most common complication when tumors metastasize to the bone, can cause depression, anxiety, and other complications in patients and even be highly debilitating to the patients' functional status and quality of life [1]. Due to the limitations of the existing treatment, many patients with bone cancer pain suffer limited pain relief and adverse side effects. Thus, understanding the potential cellular and molecular mechanisms underlying bone cancer pain is important for effectively treating these patients.

As a subtype of sigma receptor, the sigma-1 receptor is highly expressed in both neurons and glia of multiple regions within the central nervous system [2]. Extensive literature on molecular weight of sigma-1 receptor that is composed of 223 amino acids indicated a value ranging from 25 to 30 kDa. Sigma-1 receptor anchoring at the endoplasmic reticulum has been implicated in regulating inositol trisphosphate receptor- (IP3R-) mediated Ca^2+^ signaling and could translocate to the plasma or nuclear membrane once activated by the ligands [3, 4]. At the plasma membrane, the sigma-1 receptor can modulate the activation of various ion channels and receptors, such as K^+^ channels, N-methyl-D-aspartate (NMDA), dopamine, and *γ*-aminobutyric acid (GABA) receptors [5–8].

Since the discovery of the sigma-1 receptor, many preclinical studies have implicated the receptor in many diseases such as depression and neurodegenerative disease and addition [9–11]. Recently, some reports also demonstrated that sigma-1 receptor was involved in the regulation of neuropathic pain by enhancing NMDA receptors activity [12, 13]. In view of the critical role of NMDA receptors in spinal nociceptive processing of bone cancer pain, we hypothesized that the sigma-1 receptor may play an important yet unknown role in development of bone cancer pain.

In the present study, Walker 256 mammary gland carcinoma cells were implanted into the intramedullary space of the right tibia of Sprague-Dawley rats. We examined the sigma-1 receptor expression changes in the spinal cord of BCP rats. Furthermore, we tested whether intrathecal administration of the selective sigma-1 receptor antagonist BD1047 could suppress mechanical allodynia and the activation of spinal microglia as well as NR1 and the subsequent Ca^2+^-dependent signals of BCP rats.

## 2. Materials and Methods

### 2.1. Animals

Female Sprague-Dawley rats (Experimental Animal Center of Xuzhou Medical College, China) weighing 180 to 220 g were used. Animals were housed under controlled temperature (21 ± 2)°C and relative humidity (50%  ±  10%), under a 12 h light-dark cycle (light on from 08:00 to 20:00), with ad libitum access to food and water. All experimental protocols were approved by the Animal Care and Use Committee of Xuzhou Medical College. Animal treatments were performed according to the Guidelines of the International Association for the Study of Pain [14].

### 2.2. Bone Cancer Model

The Walker 256 mammary gland carcinoma cell line was prepared as previously described [15, 16]. The cells were collected and diluted to a concentration of 2 × 10^7^ cells/mL. A rat model of bone cancer pain was utilized as described previously [16]. In brief, under anesthesia with chloral hydrate (350 mg/kg, i.p.), rats were fixed, and the right tibia was prepared for surgery. The tibial plateau was exposed with minimal damage to the muscle and nerves. Walker 256 cells (1 × 10^5^ cells in 5 *μ*L of normal saline) were injected into the medullary canal through a hole drilled on the right tibia, whereas sham rats were injected with 5 *μ*L media alone. The syringe was left in place for an additional 2 min and then the injection site was closed using bone wax. The wound was sutured and treated with penicillin.

### 2.3. Drug Administration

The intrathecal injection procedure followed the method of Xu et al. in 2006 [17]. Briefly, the rats were anesthetized with sevoflurane. The lumbar region was disinfected with 75% (v/v) ethanol after hair shaving, and the intervertebral spaces were widened by placing the animal on a plexiglass tube. Next, a 29-gauge microinjection syringe needle filled with the drug was inserted in the L5-6 interspace. A brisk tail flick could be observed immediately after the needle entry into subarachnoid space. Motor function was evaluated by the observation of placing or stepping reflexes and righting reflexes at 2 minutes before a nociceptive test. Animals with signs of motor dysfunction were excluded from the experiments.

### 2.4. Mechanical Allodynia

Mechanical allodynia was assessed with Von Frey filaments (Stoelting, Wood Dale, IL) as described previously [15]. Animals were placed in separate plastic box (20 cm × 25 cm × 15 cm) on a metal mesh floor and allowed to acclimate for 30 min. The filaments were applied sequentially in an ascending order of force. Each rat was tested bilaterally. The duration of each stimulus was approximately 5 seconds with a 5-minute interval between applications. The filaments were presented perpendicularly to the plantar surface, and brisk withdrawal or paw flinching was considered as positive responses. The paw withdrawal threshold (PWT) was assessed by the “up-and-down” method [18]. All the tests were performed between 9:00 am and 12:00 am and the behavioral tests on days 5, 6, and 7 after inoculation were performed before the intrathecal injection.

### 2.5. Western Blot

For the results of Section 3.2, rats were killed on days 3, 7, 10, 14, and 21 after inoculation with cancer cells or normal saline (sham and naïve rats were killed on day 10); for the results of Sections 3.4 and 3.5, rats were killed at 2 h after BD1047 intrathecal administration on day 7. The L4-5 spinal cords of the rats were quickly extracted and stored in liquid nitrogen. All tissue samples were homogenized in lysis buffer containing PMSF and 0.02% protease inhibitor cocktail. The supernatant, after centrifugation at 12,000 revolutions per minute for 15 minutes at 4°C, was used for western blot. Equivalent amounts of protein (50 *μ*g) were separated using SDS-PAGE and transferred onto a PVDF membrane. Membranes were blocked with 5% bovine serum albumin (BSA) for 2 hours at room temperature (RT) and then washed in Tris-buffered saline with Tween 3 times for 10 minutes each. After that, membranes were incubated with primary antibodies for rabbit anti-p-NR1 (Ser896) (1 : 1000; Cell Signaling, USA), rabbit anti-p-ERK1/2 (Thr202/Tyr204) (1 : 1000; Cell Signaling, USA), rabbit anti-p-p38 (1 : 1000; Cell Signaling, USA), rabbit anti-Iba-1 (1 : 1500; Wako, Japan), rabbit anti-TNF-*α* (1 : 1000; Cell Signaling, USA), rabbit anti-GAPDH (1 : 1000; Sigma, USA), or rabbit anti-sigma-1 (1 : 200; Abcam, UK) overnight at 4°C. The membranes were incubated for 2 h with HRP-conjugated anti-rabbit secondary antibody (1 : 1500; R&D, USA). Bands were visualized using an ECL system. Data were analyzed with a Molecular Imager (ChemiDoc XRS; Bio-Rad, USA) and the associated software Quantity One-4.6.5 (Bio-Rad, USA).

### 2.6. Immunohistochemistry

At 2 h after BD1047 intrathecal administration on day 7, rats from all groups were deeply anesthetized with chloral hydrate (350 mg/kg, i.p.) and perfused intracardially with saline followed by 4% paraformaldehyde in 0.1 M phosphate buffer. L4-5 spinal cords were removed, postfixed in 4% paraformaldehyde overnight at 4°C, and then placed in a 30% sucrose solution overnight at 4°C. 25 *μ*m transverse sections were cut on a cryostat. After elimination of endogenous peroxidase activity with hydrogen peroxide and preblocking with 10% normal donkey serum and 0.3% Triton X-100 at room temperature for 2 h, the sections were incubated in primary polyclonal rabbit anti-c-Fos antibody (1 : 200; Abcam, UK) overnight at 4°C and then incubated in polymer helper (ZSGB-BIO, CN) at 37°C for 30 min and in poly-HRP anti-rabbit IgG (ZSGB-BIO, CN) at 37°C for 30 min. Finally, the sections were treated with 0.05% diaminobenzidine for 5–10 min and rinsed with PBS to end the reaction, mounted on gelatin-coated slides, air-dried, dehydrated with 30%–100% alcohol, cleared with xylene, and cover-slipped for microscopic observation (Nikon Eclipse E600, Japan). For the Iba-1 protein assay, the sections were incubated in 10% normal donkey serum and 0.3% Triton X-100 at room temperature for 2 h and then in primary polyclonal goat anti-Iba-1 antibody (1 : 200; Abcam, UK) at 4°C for 16 h. After three washes with PBS, the sections were further incubated with Alexa Fluor 488 anti-goat IgG (1 : 200; Invitrogen, USA) for 2 h at room temperature, mounted on gelatin-coated slides, and cover-slipped with a mixture of 50% glycerin in 0.01 M PBS. Images were captured with Olympus confocal microscope (Olympus FV1000, Japan) and analyzed by Image Pro-Plus 6.0 (Image Pro-Plus Kodak, USA).

### 2.7. Statistical Analysis

Statistical analysis of data was generated using GraphPad Prism 5 (GraphPad Software Inc.). All data are shown as mean ± SEM. Data from immunohistochemical analysis and western blot studies were analyzed using one-way analysis of variance (ANOVA) followed by Dunnett post hoc testing. Data of paw withdrawal threshold for mechanical allodynia testing were analyzed by two-way ANOVA followed by Bonferroni post hoc test for mechanical allodynia testing. A value of *P* < 0.05 was considered statistically significant. All the experimental testing was performed blind.

## 3. Results

### 3.1. Mechanical Allodynia Induced by Bone Cancer

**Figure 1:**
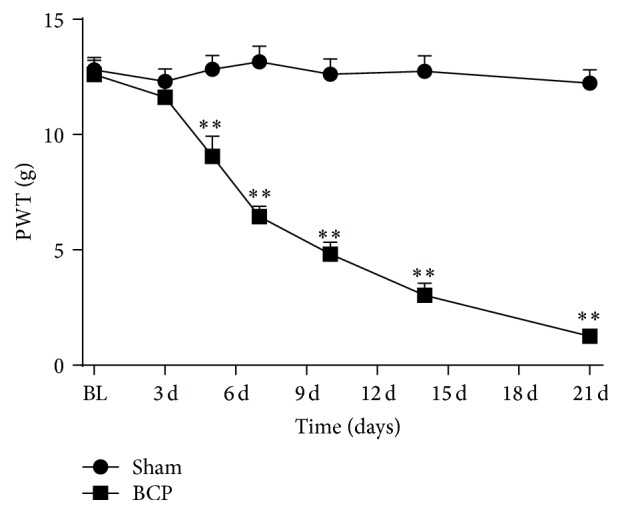
Rats with tibia tumors after Walker 256 cells inoculation displayed mechanical allodynia. The PWT progressively decreased on days 5, 7, 10, 14, and 21 (*n* = 10) after inoculation in BCP group compared with sham group. Results are given as means ± SEM. ^*∗*^ *P* < 0.05, ^*∗∗*^ *P* < 0.01 versus sham group.

All rat groups exhibited similar baseline hind paw withdrawal threshold (PWT) to mechanical stimulation (*n* = 10, *P* > 0.05). BCP rats displayed a significant decrease in PWT of the ipsilateral hind paw compared with sham rats on day 5 (*P* < 0.01; Figure 1). With the progression of bone cancer, the PWT progressively decreased in the inoculated hind paw from days 5 to 21 (*P* < 0.01; Figure 1).

### 3.2. Sigma-1 Receptor Expression Is Increased in the Spinal Cord of BCP Rats

**Figure 2:**
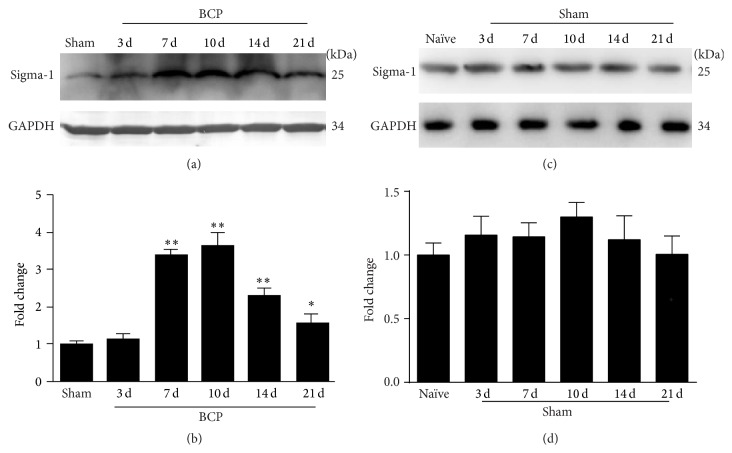
Walker 256 cells inoculation-induced sigma-1 receptor expression increased in the spinal cord. (a) Western blot analysis showed a significant upregulation of sigma-1 protein level in the spinal cord of BCP rats on days 7, 10, 14, and 21. GAPDH was used as a loading control. (b) Quantification of sigma-1 protein level in the spinal cord. (c) Western blot analysis showed no significant change of sigma-1 protein level in the spinal cord of sham rats on days 3, 7, 10, 14, and 21. GAPDH was used as a loading control. (d) Quantification of sigma-1 protein level in the spinal cord. Sigma-1 receptor levels were normalized against GAPDH (*n* = 4). Results are given as means ± SEM. ^*∗*^ *P* < 0.05, ^*∗∗*^ *P* < 0.01 versus sham group or naïve group.

Western blot analysis revealed that the expression of the sigma-1 receptor significantly increased in the spinal cord on day 7 following inoculation with Walker 256 cells compared with sham rats (*n* = 4, *P* < 0.01; Figures 2(a) and 2(b)). The protein levels further peaked on day 10 (*P* < 0.01; Figures 2(a) and 2(b)) and declined slowly from days 14 to 21 (*P* < 0.01, *P* < 0.05; Figures 2(a) and 2(b)) in BCP rats. In the spinal cord of sham rats, the expression of the sigma-1 receptor on days 3, 7, 10, 14, and 21 after surgery did not increase when compared with naïve rats (*n* = 4, *P* > 0.05; Figures 2(c) and 2(d)).

### 3.3. Blocking Sigma-1 Receptor Activation Delays Initiation of Mechanical Allodynia of BCP

**Figure 3:**
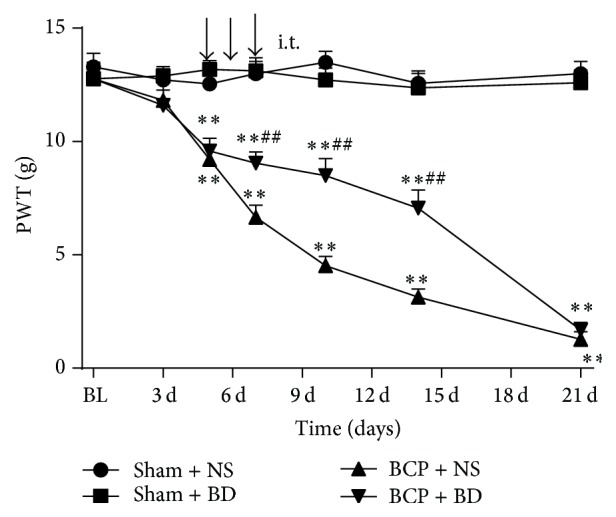
Intrathecal administration of BD1047 delayed bone cancer induced mechanical allodynia. Sigma-1 receptor antagonist BD1047 or normal saline (NS) was intrathecally injected on days 5, 6, and 7 after inoculation (*n* = 10). The PWT of BCP rats was increased after injection of BD1047 on day 7. Results are given as means ± SEM. ^*∗*^ *P* < 0.05, ^*∗∗*^ *P* < 0.01 versus sham + NS group; ^#^ *P* < 0.05, ^##^ *P* < 0.01 versus BCP + NS group.

To investigate the role of sigma-1 receptor in initiation of BCP, we measured mechanical allodynia in BCP rats after a continuous administration of the selective sigma-1 receptor antagonist BD1047 injection. We used a continuous drug administration of BD1047 (120 nmol/20 *μ*L, once a day for 3 consecutive days) from day 5 to day 7 after inoculation with Walker 256 cells. There were no significant differences in baseline PWT among all groups (*n* = 10, *P* > 0.05). Compared with normal saline-treated (NS-treated) sham group, there were no remarkable changes of PWT in BD1047-treated sham group (*P* > 0.05; Figure 3). BCP group exhibited a decrease of PWT compared with sham group on day 5. Intrathecal administration of BD1047 significantly alleviated bone cancer induced mechanical allodynia compared with NS-treated BCP group (*P* < 0.01; Figure 3).

### 3.4. Blocking Sigma-1 Receptor Activation Suppresses the Upregulation of c-Fos and p-NR1 and p-ERK in the Spinal Cord of BCP Rats

**Figure 4:**
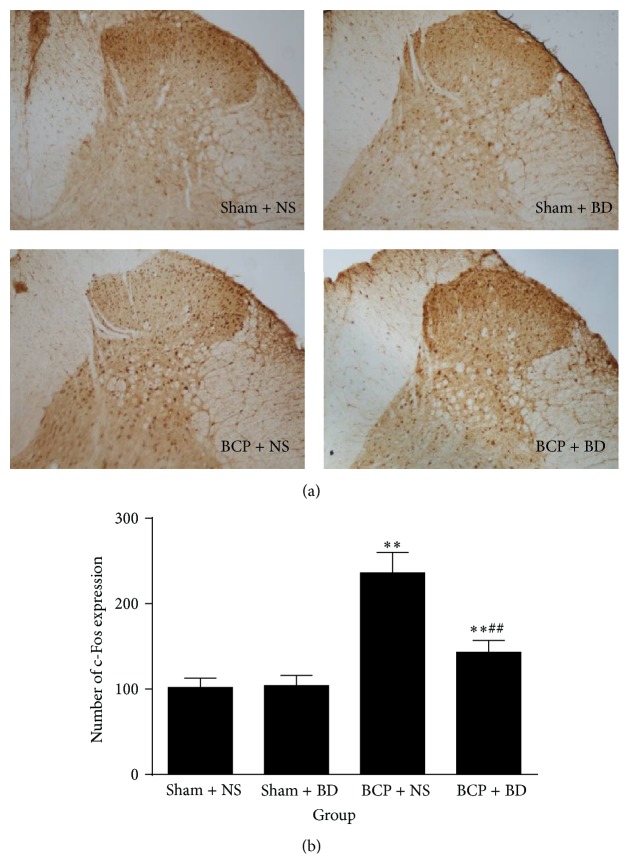
Intrathecal administration of BD1047 suppressed the upregulation of c-Fos protein expression. (a) Compared with sham rats, the c-Fos expression was strikingly increased in BCP rats in the ipsilateral spinal cord on day 7. Intrathecal injection of BD1047 provided a significant decrease of the c-Fos in BCP rats compared with NS-treated BCP group. (b) Quantification of c-Fos level in the dorsal horn (*n* = 4). Results are given as means ± SEM. ^*∗*^ *P* < 0.05, ^*∗∗*^ *P* < 0.01 versus sham + NS group; ^#^ *P* < 0.05, ^##^ *P* < 0.01 versus BCP + NS group. Magnification: 100x.

Immunohistochemistry data demonstrated that, compared with sham rats, the expression of c-Fos was strikingly increased in BCP rats in the ipsilateral spinal cord on day 7 (*n* = 8, *P* < 0.01; Figures 4(a) and 4(b)). Intrathecal administration of BD1047 from day 5 to day 7 significantly reduced the level of c-Fos protein expression in the ipsilateral spinal cord of BCP rats compared with NS-treated BCP group (*P* < 0.01; Figures 4(a) and 4(b)). There were no changes of c-Fos protein expression in NS-treated sham group and BD1047-treated sham group (*P* > 0.05; Figures 4(a) and 4(b)).

**Figure 5:**
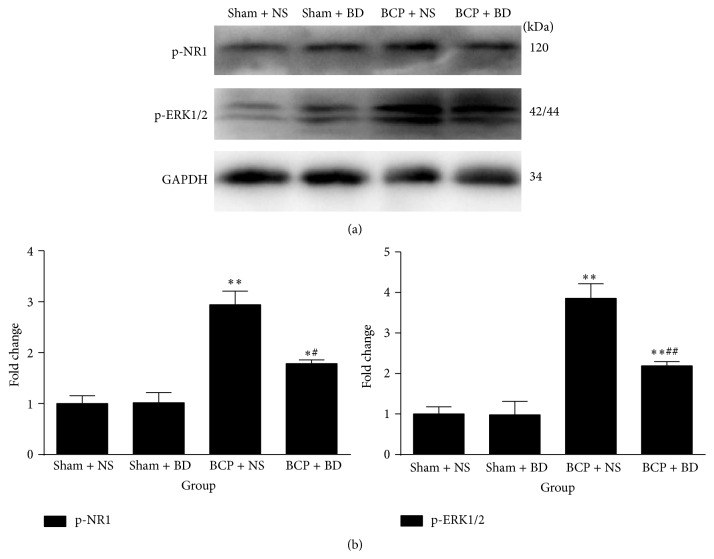
Blocking sigma-1 receptor activation suppressed BCP-induced upregulation of p-NR1 and p-ERK1/2 in the spinal cord. (a) Western blot analysis indicated a significant increase of p-NR1 and p-ERK expression in the spinal cord of BCP rats on day 7. Repetitive treatment with BD1047 significantly decreased these molecules in BCP rats compared with NS-treated BCP group. (b) Quantification of p-NR1 and p-ERK expression level in the spinal cord (*n* = 4). Results are given as means ± SEM. ^*∗*^ *P* < 0.05, ^*∗∗*^ *P* < 0.01 versus sham + NS group; ^#^ *P* < 0.05, ^##^ *P* < 0.01 versus BCP + NS group.

In addition, western blot analysis indicated that the expression of p-NR1 and p-ERK significantly increased in the spinal cord of BCP rats on day 7 compared with sham rats (*n* = 4, *P* < 0.01, *P* < 0.05, *P* < 0.01, and *P* < 0.01; Figures 5(a) and 5(b)). Repetitive treatment with BD1047 from day 5 to day 7 robustly suppressed the upregulation of these molecules in the spinal cord of BCP rats (*P* < 0.05, *P* < 0.01; Figures 5(a) and 5(b)). There were no significant differences of p-NR1 and p-ERK protein expression between NS-treated sham group and BD1047-treated sham group (*P* > 0.05; Figures 5(a) and 5(b)).

### 3.5. Blocking Sigma-1 Receptor Activation Suppresses the Upregulation of Iba-1, p-p38, and TNF-*α* in the Spinal Cord of BCP Rats

**Figure 6:**
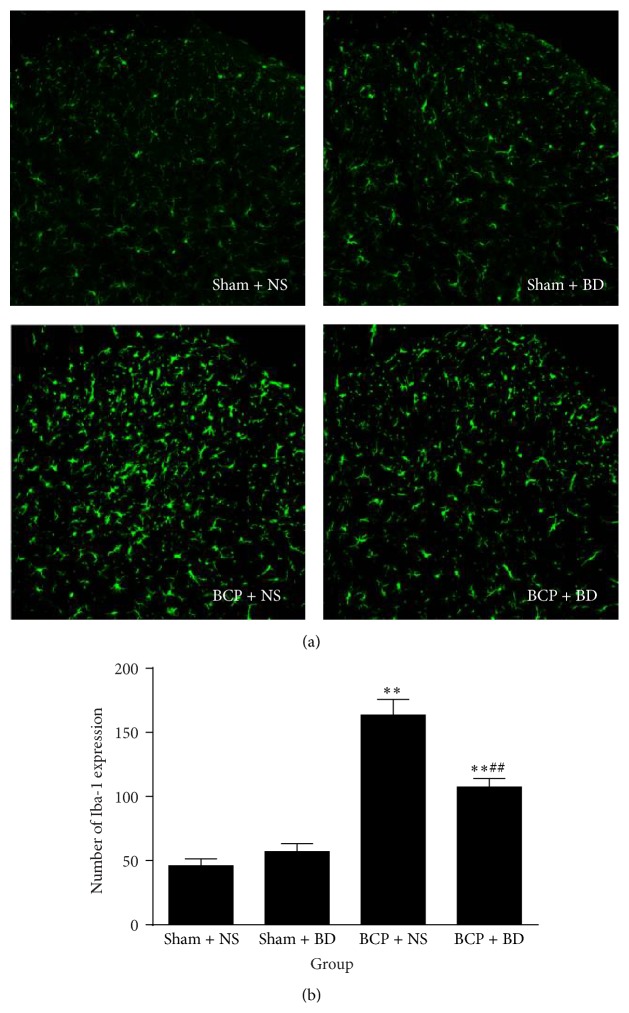
Spinal administration of BD1047 attenuated Iba-1 expression. (a) Immunohistochemistry data revealed that the expression of Iba-1 was significantly higher in BCP rats compared with sham rats in the ipsilateral spinal cord on day 7. Compared with NS-treated BCP group, BD1047-treated BCP group showed a striking decrease in the number of Iba-1 immunoreactive (IR) cells in the ipsilateral spinal cord. (b) Quantification of Iba-1 level in the dorsal horn (*n* = 4). Results are given as means ± SEM. ^*∗*^ *P* < 0.05, ^*∗∗*^ *P* < 0.01 versus sham + NS group; ^#^ *P* < 0.05, ^##^ *P* < 0.01 versus BCP + NS group. Magnification: 200x.

**Figure 7:**
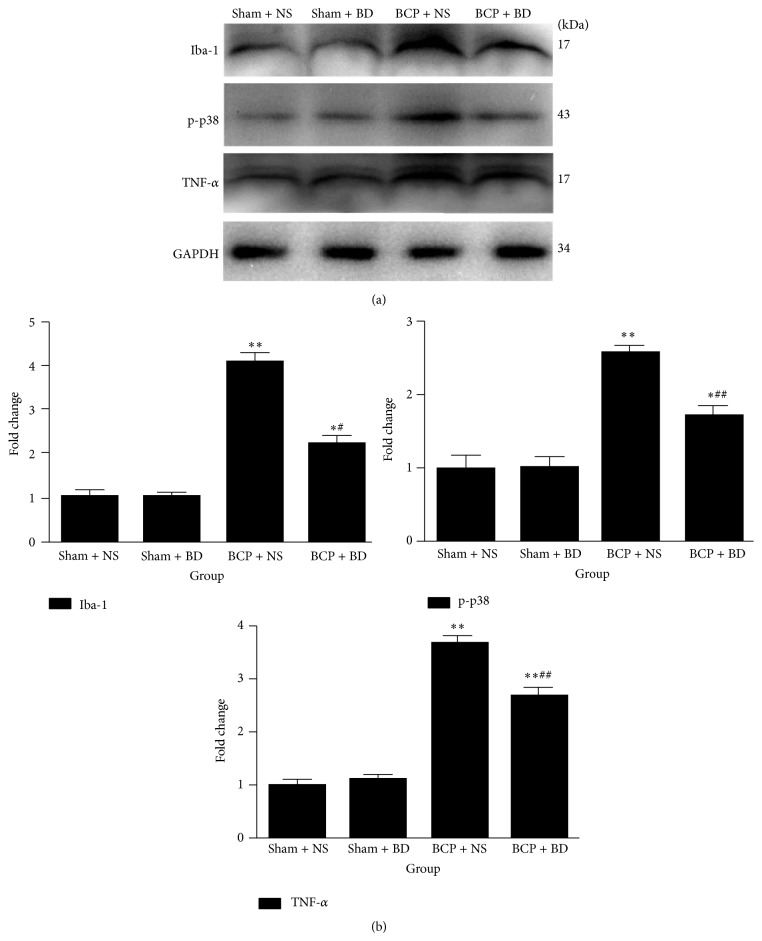
Iba-1, p38 activation. and proinflammatory cytokine expression were reduced by intrathecal administration of BD1047. (a) Western blot analysis indicated that Iba-1, p-p38, and TNF-*α* expression were higher in the spinal cord of BCP rats on day 7. Intrathecal BD1047 significantly reduced the expression of these molecules in BCP rats compared with NS-treated BCP group. (b) Quantification of Iba-1, p-p38, and TNF-*α* expression level in the spinal cord (*n* = 4). Results are given as means ± SEM. ^*∗*^ *P* < 0.05, ^*∗∗*^ *P* < 0.01 versus sham + NS group; ^#^ *P* < 0.05, ^##^ *P* < 0.01 versus BCP + NS group.

Immunohistochemistry data revealed that the expression of Iba-1 was significantly higher in BCP rats compared with sham rats in the ipsilateral spinal cord on day 7. Activated microglial cells exhibited hypertrophic morphological changes such as cell body enlargement and retraction of processes (Figures 6(a) and 6(b)). BD1047-treated BCP group showed a striking decrease in the number of Iba-1 immunoreactive (IR) cells in the ipsilateral spinal cord compared with NS-treated BCP group (*n* = 8, *P* < 0.01; Figures 6(a) and 6(b)). Consistent with immunofluorescence staining results, western blot analysis using anti-Iba-1 antibody showed that the expression level of Iba-1 was markedly increased in the spinal cord of BCP rats on day 7 compared with sham rat (*n* = 4, *P* < 0.01, *P* < 0.05; Figures 7(a) and 7(b)). Repetitive treatment with BD1047 from day 5 to day 7 robustly suppressed the upregulation of Iba-1 in the spinal cord of BCP rats (*P* < 0.05; Figures 7(a) and 7(b)).

Western blot analysis also showed that Walker 256 cells implantation induced the upregulation of p-p38 and TNF-*α* expression in the spinal cord of BCP rats compared with sham rats on day 7 (*n* = 4, *P* < 0.01, *P* < 0.05, *P* < 0.01, and *P* < 0.01; Figures 7(a) and 7(b)). Compared with NS-treated BCP group, there was a significant decrease in the level of these molecules in BD1047-treated BCP group (*P* < 0.01; Figures 7(a) and 7(b)). Iba-1, p-p38, and TNF-*α* protein expression in NS-treated sham group and BD1047-treated sham group showed no significant differences (*P* > 0.05; Figures 7(a) and 7(b)).

## 4. Discussion

The results of our study demonstrated that spinal sigma-1 receptor expression was upregulated in BCP rats and declined slowly from days 14 to 21 after surgery. Intrathecal administration of BD1047 significantly suppressed the initiation of mechanical allodynia and the spinal c-Fos expression of BCP rats. Moreover, blockade of sigma-1 receptor prevented the spinal upregulation of p-NR1 and p-ERK. In addition, our results indicated that there may be a potential relationship between the activation of sigma-1 receptor and microglia. The upregulation of Iba-1, p-p38 MAPK, and TNF-*α* expression were affected by BD1047 administration.

Previous studies have demonstrated that spinal cord sigma-1 receptor expression is upregulated under conditions of neuropathic pain [13, 19]. Our western blot data indicated that sigma-1 receptor expression was upregulated from days 7 to 21 in the spinal cord following Walker 256 cells inoculation, peaked on day 10, and declined slowly from days 14 to 21. The time course of the upregulated spinal sigma-1 receptor was not completely consistent with the development of BCP behavioral response. Thus we guess that spinal sigma-1 receptor plays a pivotal role only in the early stage of BCP.

It has been suggested that neurosteroids including pregnenolone and dehydroepiandrosterone sulfate (DHEAS) are endogenous ligands for sigma-1 receptor [20]. Several studies have reported that the concentration of spinal neurosteroids was significantly increased in neuropathic pain rats [21, 22]. Administration of DHEAS facilitated the induction of mechanical allodynia in neuropathic pain rats and leaded to pain hypersensitivity in naïve rats; both the pronociceptive effects of DHEAS could be blocked by BD1047 [23, 24]. Based on these findings, we believe that the increasing endogenous neurosteroids in the spinal cord induce pain hypersensitivity by activating sigma-1 receptor.

NMDARs are composed of three related families of subunits: NR1, NR2, and NR3 [25]. All functional NMDARs include at least one NR1 subunit and NR1 is required for receptor activity [26]. NMDARs are phosphorylated and dephosphorylated by a variety of kinases [25]. Once activated, NMDARs produce influx of Ca^2+^ and thus increase cytosolic concentration of Ca^2+^ in dorsal horn neurons. In turn, intracellular Ca^2+^ activates Ca^2+^-dependent second messengers including extracellular signal-regulated kinase (ERK) and calcium/calmodulin-dependent kinase II (CaMKII) that ultimately contribute to pain hypersensitivity [27, 28]. In the pain research field, c-Fos and p-ERK have been extensively used as the marker for the activation of nociceptive neurons and both of them are implicated in pain facilitation [29]. Previous studies reported that intrathecal injection of BD1047 blocked both mechanical allodynia and the increase in spinal NR1 expression [13, 30], which is in concordance with our data. We demonstrated that BD1047 not only attenuated behavioral hypersensitivity but also decreased BCP-induced p-NR1, p-ERK, and c-Fos expression in the spinal cord. Therefore, it is assumed that direct blockage of spinal neurons may be, at least in part, a potential target for pharmacological intervention with systemic injection of BD1047.

After Walker 256 cells implantation microglia in the spinal cord become activated [31], p-p38 expression which is exclusively expressed by microglia is markedly increased [32, 33]. The activation of p38 in spinal microglia results in increased synthesis and release of the IL-1*β*, TNF-*α*, and BDNF [34–38]. Both p-p38 MAPK and microglia-released mediators contribute to pain hypersensitivity in the spinal cord of BCP rats [38–41]. It has been reported that microglia in the central nervous system express high levels of sigma-1 receptor [42]. Pretreatment with BD1047 attenuated MAPKs and MCP-1 production induced by sigma-1 receptor agonist cocaine in BV-2 cells [43]. Moreover, intrathecal injection of BD1047 reduced the CCI-induced increase in p-p38 MAPK [19]. Our studies also demonstrated that BD1047 could significantly reduce the upregulation of spinal Iba-1, p-p38, and TNF-*α* induced by BCP. These results indicate that sigma-1 receptor is implicated in pain facilitation through the activation of spinal microglia. Recently, Hall et al. reported that administration of the nonselective sigma-1 receptor agonist DTG significantly decreased the production of TNF-*α* evoked by LPS in microglia [44]. Ruscher et al. concluded that treatment with the specific sigma-1 receptor agonist SA4503 after MCAO did not affect the increased levels of TNF-*α* [45]. These discrepancies might not be contradictory and could be explained by different modes of the compounds actions and animal models. DTG is an unspecific sigma receptor agonist with similar binding affinity to the sigma-1 receptor and the sigma-2 receptor. Therefore, further studies are needed to investigate whether the decreased expression of TNF-*α* is mediated by activation of the sigma-2 receptor. In addition, the treatment windows and mechanisms between models of middle cerebral artery occlusion (MCAO) and bone cancer pain (BCP) are different.

## 5. Conclusions

This study demonstrates that pharmacological blockade of sigma-1 receptor by i.t. administration of sigma-1 antagonist BD1047 significantly attenuates nociceptive responses to mechanical stimulation induced by Walker 256 cells implantation via the synergistic inhibition of neuronal NMDARs and microglia activation. Thus, these results suggest a potential therapeutic use of sigma-1 receptor antagonists for the clinical management of bone cancer pain.
